# The combined value of executive functions and self-regulated learning to predict differences in study success among higher education students

**DOI:** 10.3389/fpsyg.2023.1229518

**Published:** 2023-11-18

**Authors:** Diane Marcia Manuhuwa, Mirjam Snel-de Boer, Debbie Jaarsma, Joke Fleer, Jan Willem De Graaf

**Affiliations:** ^1^School of Applied Psychology and (International) Human Resource Management, Program Employability Transition, Section Inclusive Society, Saxion University of Applied Sciences, Deventer, Netherlands; ^2^Faculty of Veterinary Medicine, Utrecht University, Utrecht, Netherlands; ^3^Section Health Psychology, Department of Health Sciences, University Medical Center Groningen, University of Groningen, Groningen, Netherlands

**Keywords:** executive functions, self-regulated learning, study success, academic success, higher education, student, structural equation modeling

## Abstract

**Introduction:**

Self-regulated learning (SRL) has traditionally been associated with study success in higher education. In contrast, study success is still rarely associated with executive functions (EF), while it is known from neuropsychological practice that EF can influence overall functioning and performance. However some studies have shown relationships between EF and study success, but this has mainly been investigated in school children and adolescents. EF refer to higher-order cognitive processes to regulate cognition, behavior, and emotion in service of adaptive and goal-directed behaviors. SRL is a dynamic process in which learners activate and maintain cognitions, affects, and behaviors to achieve personal learning goals. This study explores the added value of including EF and SRL to predict study success (i.e., the obtained credits).

**Methods:**

In this study, we collected data from 315 first-year psychology students of a University of Applied Sciences in the Netherlands who completed questionnaires related to both EF (BRIEF) and SRL (MSLQ) two months after the start of the academic year. Credit points were obtained at the end of that first academic year. We used Structural Equation Modeling to test whether EF and SRL together explain more variance in study success than either concept alone.

**Results:**

EF explains 19.8% of the variance, SRL 22.9%, and in line with our hypothesis, EF and SRL combined explain 39.8% of the variance in obtained credits.

**Discussion:**

These results indicate that focusing on EF and SRL could lead to a better understanding of how higher education students learn successfully. This might be the objective of further investigation.

## 1 Introduction

Executive functions (EFs) strategies, i.e., strategies that help students learn new content or solve problems are vital in developing lifelong learning skills. However, up to now, most educational research has focused on self-regulated learning (SRL) to explain successful learning and study success (e.g., Hayat et al., 2020; Moghadari-Koosha et al., 2020; Sun et al., 2023). In contrast, EF is mainly approached from a neuropsychological and clinical perspective, focusing on EF dysfunction and related educational problems (e.g., Meltzer et al., 2018, pp. 109–141; Dijkhuis et al., 2020), and it is hardly studied in ecological settings (i.e., standard learning settings). The studies conducted on EF in the educational context have primarily focused on children and adolescents (e.g., Diamond and Ling, 2016; Pascual et al., 2019; Zelazo and Carlson, 2020), not on young adults in higher education. To our best knowledge, EF and SRL have yet to be examined in combination within the context of higher education. Therefore, this study explores the relationship between EF and SRL and the extent to which they impact students’ study success in higher education.

Executive function and SRL originate from two paradigms, respectively founded in neuropsychology and based on educational research. Both have their methods, tests, and language. Researchers generally base their research on one of two perspectives. Nonetheless, both concepts have been associated with successful studying and study success in young adults (e.g., Garner, 2009; Musso et al., 2019; Pinochet-Quiroz et al., 2022) and are essential to a broader understanding of student’s ability to learn. The following section describes the definitions, similarities and differences between EF and SRL.

Self-regulated learning is about students becoming masters of their learning process which implies being able to adopt the most appropriate strategy for a learning task to be developed (Zimmerman, 2015; Dent and Koenka, 2016). SRL is generally considered a dynamic, cyclical process consisting of different phases and sub-processes of learning (Panadero, 2017). One of the most used and well-operationalized SRL models states that the cyclical process contains the following phases: (1) forethought, planning, and activation; (2) monitoring; (3) control; and (4) reaction and reflection (Pintrich, 2004). Each phase has four different areas for regulation: cognition, motivation/affect, behavior, and context.

Executive functions can be defined as a set of cognitive processes, partially independent and involved in top-down control of behavior, emotion, and cognition (Baggetta and Alexander, 2016; Nigg, 2017). EF refer to the most basic level of behavioral analyses or the neuropsychological level (De la Fuente et al., 2022). EF are effortful and invoked when automatic responses and routines do not work. This mainly happens in novel, complex, or otherwise challenging situations (Miller and Cohen, 2001; Barkley, 2012; Diamond, 2013). Therefore, EF are critical in learning, study success, and flexible behavior (Denckla and Mahone, 2018, p. 6).

Executive functions are a multidimensional concept; the literature describes several classifications of EF. Baggetta and Alexander (2016) found 39 different components or processes of EF in their review, with three core EF being the most commonly mentioned in the 106 studies they examined, i.e., inhibition (68%), working memory (35%), and cognitive flexibility (31%):

1. Inhibition (inhibitory control, including self-control or behavioral inhibition; or interference control, including selective attention and cognitive inhibition). This is the ability to control one’s attention or inhibit dominant or automatic behavior, responses, thoughts, and emotions (e.g., Baggetta and Alexander, 2016). For example, being able to study for a more extended time without being distracted.
2. Working memory. This ability is described as keeping the information in mind and working with it (e.g., Baddeley, 2010; Diamond, 2013). For example, reading a textbook, remembering what you read, and coming up with examples from your own experience that relate to what is described in the learning materials.
3. Cognitive (or mental) flexibility (or set-shifting). This refers to the ability to literally and figuratively change perspective, remove irrelevant information and retrieve new information, think differently, or change your behavior (e.g., Diamond, 2013; Nigg, 2017). Essentially, it is about adapting to a changed situation. For instance, while working on a group assignment in an (interdisciplinary) team, being able to put yourself in someone else’s perspective.

Combining these core “lower-order” processes creates “higher-order” or complex processes such as planning, reasoning, and problem-solving (Diamond, 2013).

One of the classifications that describes both the core and complex EF and is often used in research (in both academic and clinical contexts) is that of Gioia et al. (2000). We chose this classification because of its well-operationalized EF components and its emphasis on assessing behavioral manifestations of EF in an individual’s daily life (Baggetta and Alexander, 2016). Additionally, based on this classification Gioia et al. (2000) developed the Behavioral Rating Inventory Executive Functions (BRIEF), a self-reported questionnaire that has been translated into Dutch and standardized for children (Huizinga and Schmidts, 2012) and adults (Scholte and Noens, 2011).

This classification – based on factor analyses of EF behavioral descriptions – comprises nine EF, including the three core EF described before, next to the more complex or higher-order EF “self-monitor,” “emotional control,” “initiate,” “task monitor,” “plan/organize,” and “organization of materials.”

Executive function can be conceptualized on two levels: the core EF on a cognitive level (i.e., how the brain thinks) and the core EF and complex EF on a behavioral level (i.e., how the brain thinks expressed in behavior). Both levels refer to EF; however, in studies, they are operationalized and measured differently and refer to different underlying mechanisms of EF (e.g., Barkley and Fischer, 2011; Toplak et al., 2013). Researchers hypothesize that this is why directly or task-based measured core EF hardly overlap with the indirectly or self-reported measured EF (e.g., Barkley and Fischer, 2011; Toplak et al., 2013).

An advantage of directly assessing EF is that these task-based tests better test the actual performance of a specific EF. However, these results provide information about how well the student functions in an optimal and highly structured environment and, therefore, are not easily generalized across settings (Naglieri and Otero, 2014, pp. 191–208). The advantage of a self-reported EF is its higher ecological validity because it provides information about how well the student functions in a less structured environment, such as a school or home setting (Barkley and Fischer, 2011). An assumption with self-reports is that they measure behaviors related to the cognitive processes measured by task-based measures of EF. Because we are interested in how students experience their EF in their daily settings, we use self-report questionnaires in this study to assess EF.

The same reason applies to SRL; we are interested in the students’ perceptions of their SRL in general. SRL self-reports fall under the category of “offline measures,” referring to the timing of the measurements, in this case, that the self-reports are taken before or after the task and not during the task (i.e., “online measures,” such as think-aloud protocols or systematic observations) (Veenman, 2005; Schellings, 2011). When taking an SRL self-report, the student reflects on how they usually approach the learning task, so it provides more general information than specific information about a task at that moment. Thus, it depends on the research question of which measurement instrument is most appropriate (Rovers et al., 2019).

Self-reports also have drawbacks. Paradoxically, being able to complete the self-reports requires EF of the student to reflect on past and future behaviors (Garner, 2009), suggesting that a student with weak EF will be less able to self-report.

Another issue might be that students over- or underestimate themselves and whether there is a discrepancy between their intentional behavior and what they actually demonstrate [as demonstrated for SRL by Broadbent and Poon (2015)]. Students who overstated their performance on EF performance measures also achieved significantly higher scores on self-reports (Follmer, 2021).

Rovers et al. (2019) showed that students can report – via questionnaires – relatively accurately what their general self-regulatory functioning is, while at a detailed level, they have difficulty pinpointing exact SRL strategies. They argue that the level of granularity is of influence and that different types of measurement are valuable, depending on the research question. The same kind of reasoning could apply to the practical use of these measurements. For example, the benefit of self-reporting is that students become aware of their SRL strategies, which is an intervention in itself (Panadero et al., 2016). So, if self-monitoring is the objective, self-reports are an excellent option.

Conceptually, both EF and SRL refer to higher-order (top-down) cognitive processes. However, they differ in the context in which they are applied. EF are essential for navigating everyday life and engaging in social interactions (Barkley, 2012). EF become active when a student faces new, complex, or challenging daily life problems, including but not limited to problems encountered in the learning environment. In those moments, the student must make decisions, resolve issues, learn from mistakes, mentally play with ideas (be creative), think before acting, resist temptations, and stay focused (Diamond, 2013). In contrast, SRL occurs specifically and exclusively in the learning context and focuses on acquiring knowledge, automating skills, and achieving learning results. SRL involves both conscious and unconscious deep processing of information, or the repetition of facts, to eventually consolidate this information in long-term memory (Wirth et al., 2020).

Another difference is that SRL strongly emphasizes motivation or the “why” someone does something and the willingness to put effort into it (Schunk and Greene, 2018), in contrast to EF, which focus more on the “how,” i.e., “how do I solve this problem or adapt to the situation?”

Studies on the *relationship between EF and SRL* suggest a partial overlap between the constructs (e.g., Garner, 2009; Effeney et al., 2013; Follmer and Sperling, 2016). In these studies, EF and SRL are – indirectly – measured via self-reports demonstrating that EF expressed on a *behavioral* level overlap partially with SRL, also expressed on a behavioral level. Moreover, it seems that, in particular, the metacognitive dimensions of SRL are associated with or coincide with EF, for instance, planning (Effeney et al., 2013; Pinochet-Quiroz et al., 2022). The ability to plan allows a student to set and achieve goals in everyday life (context of EF) and focus explicitly on prioritizing learning tasks (context of SRL). However, self-reported EF and SRL are not the same in learning environments, and when overlapping, EF appear to contribute to variability in SRL processes, and the other way around, SRL processes implicate EFs (Garner, 2009).

However, in contrast, EF appear to be more unidirectionally related to SRL when measured directly through neuropsychological tasks, i.e., meaning task-based EF mediate through SRL on academic achievement and not the other way around (e.g., Rutherford et al., 2018; Musso et al., 2019). Only the core EF (i.e., working memory, inhibition, or cognitive flexibility) are task-based measured in these cases. In other words, SRL strategies seem to employ *core* EF – on a cognitive level – to achieve learning results, which makes sense because to sustain a learning strategy, the student must focus, keep information online, avoid distractions, and be cognitively flexible in disregarding old information in favor of new information.

In summary, EF can be conceptualized and measured at a cognitive and behavioral level (typically task-based and self-reported). Self-reported EF are most likely to have a partially overlapping relationship with self-reported SRL. In contrast, task-based EF are more likely to support self-reported SRL in achieving study success, thus showing a mediating role.

Studies about *EF, SRL, and study success* are scarce, particularly in young adult students. In this population, we identified only the study by Musso et al. (2019), who investigated the coherence between EF, SRL, and study success in a group of first-year university students. They found mediating effects of EF via SRL on math performance. Musso et al. (2019) measured EF directly (i.e., measured with neuropsychological tasks) and focused solely on working memory and executive attention.

To our knowledge, no study has focused on self-reported EF and SRL together as predictors of study success in higher education. There are a few studies that have investigated the relationship between self-reported EF and study success in the context of higher education. These studies indicate that self-reported EF problems negatively affect study success (e.g., Knouse et al., 2014; Baars et al., 2015; Ramos-Galarza et al., 2020). On the other hand, numerous studies have shown a positive relationship between SRL and study success in higher education (e.g., Honicke and Broadbent, 2016; Virtanen et al., 2017; Sun et al., 2018). The added value of EF in conjunction with SRL and their explanatory value for study success still needs to be determined to investigate if the concepts combined have the potential power to improve study success. Therefore, this study investigates the following research question:

Do self-reported EF and SRL combined explain variations in study success among higher education students better than either separately? Notably, since there appears to be a reciprocal non-mediating relationship between self-reported EF and self-reported SRL, we assume two independent variables that can directly or combined affect study success.

**FIGURE 1:**
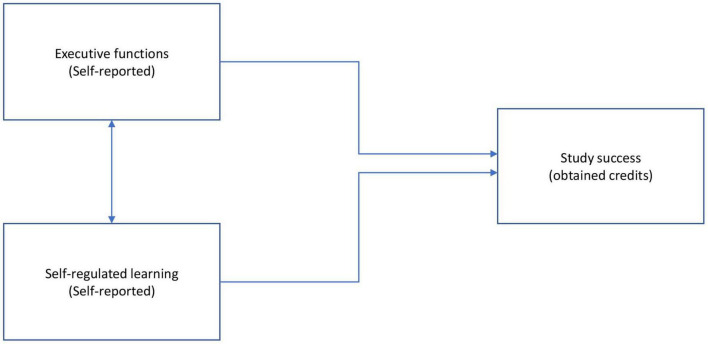
Research model of the relationship EF and SRL, and study success.

Following the research model depicted in Figure 1, we will investigate (1) the relationships between EF and SRL (measured 2 months after the start of the academic year), and study success (measured at the end of the academic year, and (2) the combined effect of EF and SRL on study success.

We hypothesize that EF and SRL combined explain statistically significantly more variance in the number of credits obtained at the end of the academic year than each construct separately.

In addition, the aim is not to examine the different dimensions of EF and SRL and their relationships. Given the inter- and intra-individual differences due to the developmental trajectory of both EF and SRL, we expect these specific dimensions to have little expressive power when looking at individual students. We expect the results to provide insight into the group. However, this picture may differ if students have been developing for 6 months or if a different group of students is involved. Therefore, we explore the concepts of EF and SRL without identifying the specific dimensions.

The COVID-19 pandemic made studying and life more challenging for students due to lockdowns and regulations (e.g., Copeland et al., 2021; Ihm et al., 2021). During this time, students may have faced a constant stream of new and complex issues, which could have impacted their EF. While not the main focus of our research, we also wanted to understand how these circumstances affected students’ self-reported EF and SRL. As such, we asked students if the lockdowns and regulations influenced how they completed our questionnaire.

## 2 Materials and methods

### 2.1 Procedures

This study was conducted following a retrospective cohort design (Ato et al., 2013). EF and SRL data for this longitudinal survey study were collected from the last week of November 2020 through the first week of December 2020 during the first-year module “Diagnostic Research Part 1 (DR1).” At the end of the academic year in July 2021, we collected the obtained credits. One of the main objectives of module DR1, is learning to conduct research. In that context, the students fill out various questionnaires to experience what participating in research entails.

The study measurements were integrated into the educational program so students could complete the online questionnaires during a lesson. All the students received their results and feedback regarding their test performances. As a follow-up, students were offered to discuss their results with the researcher, lecturer, or mentor.

In the *first week of the module*, the students were informed about the study aim and procedure during an online lecture. They were told that completing the questionnaires would take approximately 45–60 min, that participation was voluntary and confidential, and that no credits were involved. According to institutional ethical advice committee (SEAC) guidelines, informed consent was drawn and provided for signature at the start of the procedure. All students were invited to participate, but we only used the results of students who signed the informed consent for analyses.

During class in the *second week of the module*, students completed the questionnaires on first EF (Behavior Rating Inventory of Executive Function – Adult version) then SRL (Motivated Strategies for Learning Questionnaire), then descriptive questions, such as the COVID-19 control question. The completion of the questionnaires would take students approximately 30–60 min.

The credits earned at the end of the school year were retrieved from the school’s database and could be up to 60 credits.

### 2.2 Participants

This study included all first-year students of the program Applied Psychology of the University of Applied Sciences in the Netherlands. The inclusion criteria were *first-year higher education students* between *18 and 25 years*, assuming that around 25, the prefrontal cortex is mature, and the EF are optimally developed (Giedd and Rapoport, 2010). We are particularly interested in first-year students because the transition from high school to higher education impacts this group because they must learn new ways of learning and personal changes, such as living independently (Lowe and Cook, 2003; Carragher and McCaughey, 2022). We excluded student younger or older than 25 years.

A total of 484 first-year students participated in module DR1 and completed the questionnaires. Of them, 444 signed the informed consent. We excluded 129 students for various reasons (Table 1). The final sample contained 315 first-year higher education students. Table 2 includes the descriptive data.

**TABLE 1:** Overview of the selection process of the final sample size for the structural equation modeling analyses.

Number of students that completed one or both questionnaires (*n* = 484)	Number of removed respondents	Sample size
Students who did *not* sign the informed consent	40	444
Students of age (<18 and >25 years; or unknown)	99	345
Students (18 years and older) who filled out the wrong version of the BRIEF*	3	342
Students who filled out the BRIEF improbably, inconsistently and negatively**	0	342
Students who did *not* fill out the BRIEF *and* MSLQ	27	315
Final sample		315

*We provided the BRIEF-2 version for students 16 and 17 years old. However, some of the 18 years and older, who should fill out the adult-version of the BRIEF, clicked on the wrong link and completed the wrong BRIEF. We analyzed the data of the 16 and 17-year-old students in a different study.

**These are three validity scales of the BRIEF to evaluate whether the student’s answer pattern is not overly negative, inconsistent, or atypical.

**TABLE 2:** Descriptive analyses.

Variables	Mean	SD	%	*n*
Age (years)	19.80	1.73		315
**Gender**				
Male			31.7%	100
Female			67.3%	212
Different			1.0%	3
**Education before applied university (in the Netherlands)**				
Havo			54.9%	173
Vwo			7.9%	25
Mbo			30.5%	96
Hbo			5.7%	18
Other			1.0%	3

Havo and VWO are comparable to high school; Mbo compares to regular secondary vocational education; Hbo refers to higher education.

### 2.3 Measures

#### 2.3.1 Study success

Study success was measured by retrieving the number of credits earned after the first school year (including two semesters) from the university’s database.

In addition, we used two self-report measures, namely for EF and SRL, which we discuss further below.

#### 2.3.2 Behavior Rating Inventory of Executive Function – Adult version

The BRIEF-A is a self-report questionnaire to describe EF based on behaviors of adults (18–90 years) (Roth et al., 2005, 2013). This instrument has been standardized for the executive functioning of adults in everyday environments, and specifically in the Netherlands for adults aged 18–65 years (Scholte and Noens, 2011).

The BRIEF-A includes nine non-overlapping and empirical-based scales (Table 3). The nine scales are measured by 75 items about perceived EF deficits over the past month on a three-point scale (1 = never; 2 = sometimes; 3 = often). The higher the score on specific behaviors, the higher the level of perceived EF deficits.

**TABLE 3:** Reliability subscales BRIEF-A and example items.

Index (total of subscales) Subscale	Cronbach’s α	Number of items	Example item
**Behavioral regulation**			
Inhibition	0.68	8	I am impulsive
Shift	0.72	6	I am bad at change
Emotional control	0.89	10	I overreact to minor problems
Self-monitor	0.66	6	I say things without thinking
**Metacognition**			
Initiate	0.75	8	I find it challenging to start working independently
Working memory	0.77	8	I can only concentrate for a short time
Plan	0.78	10	I don’t plan tasks ahead
Task monitor	0.69	6	I make sloppy mistakes
Organization of materials	0.81	8	I don’t clean up my mess

The total raw scores of the subscales can be transformed into *T*-scores, making it possible to compare them with a representative norm group. A *T*-score of 65 or greater indicates “clinical” problems with a specific EF or a cluster of EF (the total or index scores) (Roth et al., 2005; Scholte and Noens, 2011). However, Schwartz et al. (2020) and Abeare et al. (2021) demonstrated that in some cases, particularly in clinical samples, a cut-off *T*-score of ≥80 or ≥90 demonstrates higher specificity and is a more realistic representation.

There is always a percentage of the participants unwilling or unable to complete the questionnaires credibly. Therefore, we should be aware of the symptom validity, i.e., the extent to which scores on self-reports reflect true levels of emotional distress (Larrabee, 2012). In particular, young adults (i.e., students) cannot always realistically assess their EF deficits (Toplak et al., 2013), which is demonstrated by the lack of relationship between both subjectively and objectively measured cognitive abilities, with other psychological factors believed to play a role, such as depressive symptoms (Toplak et al., 2013). Therefore, it is recommended to control for this through symptom validity testing. The BRIEF-A contains three validity scales to measure three aspects of non-credible responding (negativity, inconsistency, and infrequency) (Roth et al., 2005; Scholte and Noens, 2011).

We calculated the internal consistency of the BRIEF-A and its component subscales. The analysis yielded acceptable internal consistency reliability (Table 3).

#### 2.3.3 Motivated Strategies for Learning Questionnaire

The MSLQ assesses SRL (Duncan and McKeachie, 2015). It is a self-report questionnaire designed to assess students’ motivational orientations and the use of different learning strategies. Self-reports have proven reliable and valid instruments for gaining general insight into students’ SRL (Rovers et al., 2019).

The questionnaire consists of a motivational and a learning strategies scale, consisting of respectively six and nine subscales (Table 4).

**TABLE 4:** Reliability subscales MSLQ and example items.

Index (total of subscales) Subscale	Cronbach’s α	Number of items	Example item
**Motivational beliefs**			
Intrinsic goal orientation	0.69	4	The most satisfying thing for me in this course is trying to understand the content as thoroughly as possible
Extrinsic goal orientation	0.69	4	If I can, I want to get better grades in this class than most of the other students
Task value	0.87	6	I like the subject matter of this course
Control of learning beliefs	0.67	4	If I try hard enough, then I will understand the course material
Self-efficacy for learning and performance	0.93	8	I expect to do well in this class
Test anxiety	0.86	5	When I take tests I think of the consequences of failing
**Learning strategies**			
Rehearsal	0.77	4	I memorize keywords to remind me of important concepts in this class
Elaboration	0.74	6	When reading for this class, I try to relate the material to what I already know
Organization	0.79	4	I make simple charts, diagrams, or tables to help me organize course material
Critical thinking	0.74	5	I try to play around with ideas of my own related to what I am learning in this course
Metacognitive self-regulation	0.77	11	I try to think through a topic and decide what I am supposed to learn from it rather than just reading it over when studying
Time and study environment	0.76	8	I make good use of my study time for this course
Effort regulation	0.72	4	I work hard to do well in this class even if I don’t like what we are doing
Peer learning	0.50	3	I try to work with other students from this class to complete the course assignments
Help-seeking	0.73	4	I ask the instructor to clarify concepts I don’t understand well

The subscales of the MSLQ demonstrate moderate to good internal consistency reliability, except for Peer learning, which was poor (Table 4). This may be because this scale contains the least number of items, namely three items, or that it is a different population than the one with which the MSLQ was validated.

#### 2.3.4 Impact of COVID-19

To gain insights into the impact of the COVID-19 pandemic, we asked the students if the lockdowns and regulations influenced how they filled out the questionnaire. Response options were as follows*: 1* = *I am more negative/I experience more problems; 2* = *I do not act differently than before COVID-19; 3* = *I am more optimistic/I notice challenges.*

### 2.4 Statistical analyses

Pearson’s correlation coefficients are calculated to explore the relationships between the BRIEF-A and MSLQ subscale scores. According to Cohen (1992), <0.3 means a weak correlation, 0.3–0.5 is a moderate correlation, and 0.5 or higher is a strong correlation effect.

To test our hypothesis, structural equation modeling (SEM) was conducted. SEM is a statistical method that uses various models to test hypothesized relationships among observed variables (Schumacker and Lomax, 2015). The following standard model fit indices were used: Chi-square-test (χ^2^), standardized root mean residual (SRMR) confirmatory fit index (CFI), and root mean square error of approximation (RMSEA).

A non-significant χ^2^ test is considered as good. In contrast, a large and significant χ^2^ test indicates a big discrepancy and, thus, a poor fit between the model and original data (Hu and Bentler, 1999). Because the χ^2^ becomes increasingly unreliable when the sample size is more significant than 250 (Byrne, 2006), the statistic χ^2^ divided by its degrees of freedom (df) is used (Bollen, 1989), where a ratio >2.00 represents an inadequate fit (Byrne, 2006).

A value less than 0.08 is considered a good fit for the SRMR, an absolute measure indicating zero as a perfect fit. The SRMR has no penalty for model complexity (Hu and Bentler, 1999). CFI values >0.90 and 0.95 indicate acceptable and excellent fit, respectively, and RMSEA values <0.06 and <0.08 indicate a good to acceptable fit (Hu and Bentler, 1999).

A rule of thumb of confirmatory factor analyses (CFA)/SEM is often a ratio of cases to free parameters, or N:p, namely at least 10:1 to 20:1 (e.g., Schumacker and Lomax, 2015; Kline, 2016). In our study, we sufficiently achieve the minimum ratio of 10:1 with our sample of 315 cases and 27 variables.

The first step of SEM involves specifying a set of latent variables and their relations. We tested the constructs (EF and SRL) with CFA, using maximum likelihood (ML) estimation, with AMOS 26.0, to identify the measurement model for study success. This resulted in a model with low CFI (0.64) and high multicollinearity.

Therefore, we conducted an exploratory factor analysis (EFA) per theoretical construct. As a result, the latent variables were established, and the construct –“Peer learning”- was removed due to low reliability (α = 0.50), inadequate convergent validity (factor loadings were 0.27, 0.44, and 0.87) and the fact that the construct loads with Help-Seeking (for an overview of the included items, see Supplementary Tables 1, 3, 4). Also before and after the EFA some of the other constructs had a suboptimal reliability (e.g., Self-Monitor and Anger Outbursts). Although a Cronbach’s alpha of minimally 0.7 is preferable, a value of 0.6 is acceptable (Hajjar, 2018). The observable measures for the latent variables were partially adjusted (for an overview of the removed items, see Supplementary Tables 2–4).

Again, a CFA was conducted, showing a reasonable amount of multicollinearity, but the model fit measures are between acceptable margins (χ^2^/df = 1.55; SRMR = 0.06; RMSEA = 0.04; CFI = 0.85).

The second step comprises creating a SEM, including all the defined latent variables to test our hypothesis. This Model 1 combined all the variables to test how EF and SRL would explain the total variance in study success. Again, we expected that not all variables would be (equally) significantly correlated and contribute to the variance of study success.

Additionally, two models were created and tested to establish the contribution of SRL and EF separately to gain insights into the differences between the concepts separately and combined. Model 2 comprised all the SRL latent variables, and Model 3 the EF latent variables. A Chi-square difference test statistic was used to measure the differences between the models (where *p* < 0.05 means a significant difference).

## 3 Results

The study was conducted as planned with no significant details in implementation. The results will answer the research question of the combined value of EF added to SRL to better understand the differences in study success. We hypothesize that EF and SRL combined explain statistically significantly more variance in the number of credits obtained at the end of the academic year than each construct separately.

First, we provide descriptive data, then discuss the correlations between the different constructs (EF and SS, SRL and SS, and EF and SRL) and finally, test the hypothesis.

### 3.1 Descriptive statistics

The students’ total BRIEF-A *raw* scores ranged between 75 and 178. The average was 118.01 (SD = 19.10). Their average *normative* scores fell in the range of “normal or average” level of perceived EF deficits (*T*-score between 30 and 59) to “clinical (*T*-scores 65 and higher).” Approximately 17% of the student population was in the subclinical range (defined as a *T*-score of 60–64), whereas 20% of the students perceived EF deficits in the clinical range (*T*-score ≥65). It is expected that for some EF scales a cut-off score of *T* ≥ 80 may be more valid, for instance, “working memory” (Abeare et al., 2021). So, the percentage of students that realistically report “clinical” EF deficits, might be lower than 20%.

The MSLQ has no specific cut-off scores. The average total score of motivated strategies of students was 5.06 (SD = 0.57) on a scale of 1–7. The average total score of learning strategies was 4.49 (SD = 0.65) on a scale of 1–7.

Finally, the obtained credits after one school year ranged from 0 to 60, with 60 points being the maximum possible. The average was 49.03 (SD = 15.54).

### 3.2 The correlations between executive functions, self-regulated learning, and study success

We conducted correlation analyses to examine the relationships between EF-SRL, EF-study success, and SRL-study success. Table 5 shows the relationships between EF and SRL. We found weak correlations (range *r* = −0.23 to *r* = −0.26) among the composite scores of the self-reported measures: between EF behavioral and metacognitive indices and SRL motivational beliefs index, and the EF indices and SRL learning strategies index.

**TABLE 5:** Pearson product-moment-correlations between self-regulated learning and executive functions.

EF indices and subscales SRL indices and subscales	Behavioral index	Metacognition index	Inhibit	Shift	Emotional control	Self-monitor	Initiate	Working memory	Plan/ organize	Task monitor	Organizing materials
Motivational beliefs	−0.23**	−0.26**									
2. Intrinsic goal orientation			−0.11*	−0.03	−0.06	0.01	0.00	−0.16**	−0.09	−0.06	−0.06
3. Extrinsic goal orientation			−0.15**	−0.10	0.12*	−0.19**	0.03	−0.23**	−0.12*	−0.15**	−0.13*
4. Task value			−0.09	−0.06	−0.13*	−0.08	−0.04	−0.18**	−0.08	−0.08	−0.09
5. Control beliefs			−0.00	0.03	−0.18**	−0.12*	−0.03	0.00	−0.01	0.00	−0.18**
6. Self-efficacy			−0.19**	−0.04	−0.23**	−0.12	−0.12	−0.30**	−0.22*	−0.28**	0.00
7. Test anxiety^1^			0.23**	0.04	0.35**	0.41**	0.04	−0.22**	0.21*	0.23**	0.16*
Learning strategies	−0.09	−0.24**									
8. Rehearsal			−0.12*	−0.06	0.06	0.12*	0.06	−0.17**	−0.11	−0.14*	−0.12*
9. Elaboration			−0.14*	−0.07	−0.08	0.05	−0.05	0.23**	−0.18**	−0.20*	−0.17**
10. Organization			−0.13*	−0.07	0.07	0.15**	0.00	−0.25**	−0.09	−0.22**	−0.16**
11. Critical thinking			0.05	0.10	−0.07	−0.00	0.01	0.09	0.05	0.05	0.05
12. Metacognitive self-regulation			−0.14**	−0.16**	−0.12*	−0.02	−0.12*	−0.24**	−0.20**	−0.23**	−0.19**
13. Time and study environment			−0.37**	−0.34**	−0.11*	−0.09	−0.24**	−0.51**	−0.34**	−0.51**	−0.41**
14. Effort regulation			−0.36**	−0.35**	−0.11*	−0.09	−0.24**	−0.52**	−0.34**	−0.50**	−0.40**
15. Peer learning			−0.08	−0.01	−0.10	0.01	0.04	−0.07	−0.03	−0.12*	0.10
16. Help-seeking			−0.11*	−0.08	−0.08	−0.01	−0.02	−0.16**	−0.10	−0.21**	−0.14*

The behavioral index score includes inhibit, shift, emotional control, and self-monitor. The metacognitive index score includes the initiate, working memory, plan/organize, task monitor, and organization of materials. Motivation scales refer to the total of the subscales below. The same is true for learning strategies. Test anxiety is reversed; a higher score refers to more test anxiety which implies more problems with studying.

^1^*Correlation is significant at 0.05 level (two-tailed).

**Correlation is significant at 0.01 level (two-tailed).

At the level of subscales, there are many weak to strong negative correlations between SRL subscales and EF subscales. The directions of the significant correlations indicate that students who report more EF problems also report using fewer SRL strategies.

Table 6 describes the correlations between EF and study success. We found weak but significant correlations between study success and six EF subscales (range *r* = −0.12 to *r* = −0.24). These (weak) correlations suggest that an increase in EF problems is associated with less study success.

**TABLE 6:** Pearson product-moment-correlations between executive functions and executive functions and study success (obtained credits).

Variable	Mean	SD	Range	1	2	3	4	5	6	7	8	9	10	11	12
1. Study success	49.03	15.54	0–60	–											
2. Executive functions total	116.51	19.00	69–207	−0.21**	–										
3. Behavioral index	47.93	8.61	30–90	−0.02	0.70**	–									
4. Metacognition index	68.58	14.35	39–117	−0.27**	0.90**	0.33**	–								
5. Inhibit	13.63	2.88	8–24	−0.18**	0.53**	0.62**	0.33**	–							
6. Shift	9.78	2.52	6–18	0.07	0.51**	0.71**	0.25**	0.21**	–						
7. Emotional control	15.89	4.67	10–30	0.08	0.50**	0.82**	0.17**	0.18**	0.56**	–					
8. Self-monitor	8.63	1.97	6–18	−0.09	0.45**	0.61**	0.23**	0.56**	0.22**	0.23**	–				
9. Initiate	14.5	3.36	8–24	−0.24**	0.66**	0.43**	0.62**	0.40**	0.36**	0.24**	0.24**	–			
10. Working memory	14.22	3.32	8–24	−0.12**	0.61**	0.54**	0.49**	0.54**	0.41**	0.28**	0.37**	0.55**	–		
11. Plan/organize	16.46	3.75	9–27	−0.20**	0.61**	0.45**	0.53**	0.48**	0.33**	0.21**	0.34**	0.69**	0.66**	–	
12. Task monitor	10.91	2.14	6–18	−0.19**	0.59**	0.48**	0.49**	0.58**	0.26**	0.20**	0.45**	0.60**	0.60**	0.68**	–
13. Organization of materials	13.99	3.57	8–24	−0.16**	0.44**	0.30**	0.41**	0.37**	0.12*	0.18**	0.21**	0.48**	0.40**	0.51**	0.55**

A higher BRIEF score refers to more self-reported problems with executive functions. The “Executive functions total” is the total score of all executive functions (5–13). The behavioral index score includes inhibit, shift, emotional control, and self-monitor. The metacognitive index score includes initiate, working memory, plan/organize, task monitor, and organization of materials.

*Correlation is significant at 0.05 level (two-tailed).

**Correlation is significant at 0.01 level (two-tailed).

Table 7 describes the correlations between SRL and study success. The correlation analysis between SRL and study success resulted in weak significant correlations between study success and six SRL subscales (range *r* = −0.11 to *r* = 0.21). Overall, these findings suggest that an increase in applying SRL is associated with more study success.

**TABLE 7:** Pearson product-moment-correlations between self-regulated learning, and self-regulated learning and study success (obtained credits).

Variable	Mean	SD	1	2	3	4	5	6	7	8	9	10	11	12	13	14	15
1. Study success	49.03	15.54	–														
Motivation scales	5.06	0.57															
2. Intrinsic goal orientation	5.03	0.90	0.10	–													
3. Extrinsic goal orientation	4.82	1.09	0.17**	0.22**	–												
4. Task value	5.05	0.95	0.00	0.59**	0.30**	–											
5. Control beliefs	5.46	0.81	−0.05	0.27**	0.09	0.36*	–										
6. Self-efficacy	5.53	0.77	0.11	0.44**	0.26**	0.40**	0.52**	–									
7. Test anxiety^1^	3.50	1.41	−0.06	−0.07	0.30*	0.01	−0.19**	−0.30**	–								
Learning strategies	4.49	0.65															
8. Rehearsal	4.38	1.25	−0.04	0.29**	0.27**	0.36**	−0.02	0.10	0.16**	–							
9. Elaboration	4.94	0.88	0.13*	0.46**	0.30**	0.48**	0.22**	0.36**	0.08	0.58**	–						
10. Organization	4.36	1.24	0.03	0.28**	0.36**	0.32**	0.00	0.14*	0.23**	0.70**	0.65**	–					
11. Critical thinking	3.97	0.98	−0.18*	0.30**	0.10	0.21**	0.06	0.11	0.08	0.15**	0.27**	0.21**	–				
12. Metacognitive self-regulation	4.42	0.76	0.06	0.46**	0.25**	0.42**	0.19**	0.30**	0.02	0.52**	0.67**	0.56**	0.39**	–			
13. Time and study environment	5.19	0.80	0.20**	0.26**	0.20**	0.30**	0.01	0.39**	−0.15*	0.21**	0.31**	0.26**	−0.13*	0.31**	–		
14. Effort regulation	5.03	0.96	0.21**	0.29**	0.23**	0.27**	−0.05	0.33**	−0.07	0.21**	0.31**	0.28**	−0.12*	0.31**	0.76**	–	
15. Peer learning	3.89	1.18	−0.04	0.23**	0.12*	0.18**	0.00	0.15**	0.02	0.20**	0.37**	0.29**	0.31*	0.39*	0.10	0.06	–
16. Help-seeking	4.21	1.22	0.11*	0.18*	0.10	0.04	−0.10	0.08	−0.02	0.24**	0.39**	0.29**	0.07	0.34**	0.26**	0.20**	0.53**

^1^Test anxiety is reversed; a higher score refers to more test anxiety which implies more problems with studying.

*Correlation is significant at 0.05 level (two-tailed).

**Correlation is significant at 0.01 level (two-tailed).

To sum up, we found significant correlations between all the constructs.

### 3.3 Hypothesis test of the explanatory model of variances in study success

**FIGURE 2:**
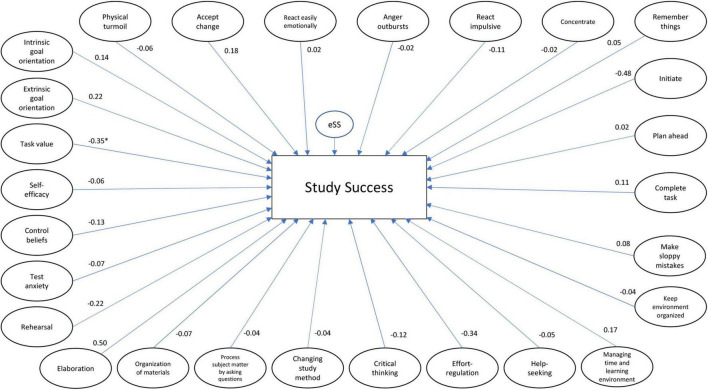
Total model, including all the latent variables of EF (starting above left “physical turmoil to keep environment organized”) and SRL variables, standardized betas, and *p*-values. *p < 0.05.

To test our hypothesis, we evaluated the model fit through SEM after performing confirmatory and exploratory analyses. The model shown in Figure 2 was tested.

The total model without restrictions fit the data well according the Hu and Bentler (1999) thresholds (χ^2^/df = 1.53; SRMR = 0.06; RMSEA = 0.04; CFI = 0.84), except for the CFI.

The CFI does not reach the preferred threshold of a minimal 0.90, meaning that the hypothesized model may not fit the observed data as well as is preferred (Van Laar and Braeken, 2021). Nevertheless, a model with a CFI value below 0.90 can be interpreted if the other measures meet the stated requirements – which is the case (Marsh et al., 2004). Another consideration is that the CFI might have dropped because our model is large and complex (Pat-El et al., 2013), and Hu and Bentler’s cut-off values may be too stringent in these cases (Marsh et al., 2004, 2005). Specifically, Marsh (2007, p. 785) states that “It is almost impossible to get an acceptable fit (e.g., CFI, TLI > 0.9; RMSEA < 0.05) for even ‘good’ multifactor rating instruments when analyses are done at the item level and there are multiple factors (e.g., 5–10), each measured with a reasonable number of items (e.g., at least 5–10/per scale) so that there are at least 50 items overall” which is the case in our study. Therefore, we continued testing the model, demonstrating that the total model explains 40.1% of the variance in obtained credits (Table 8).

**TABLE 8:** Structural equation model (SEM) of EF and SRL, and EF separately to SRL.

	*χ* ^2^	df	*χ*^2^/df	SRMR	RMSEA [CI]	CFI	AIC	Model comparison	df	Δ CMIN	*p*
Model 1: total	6530.21**	4278	1.53	0.06	0.04 [0.04, 0.04]	0.84	7676.209				
Model 2: EF	1090.92**	743	1.47	0.06	0.04 [0.03, 0.04]	0.93	1410.922	2 vs. 1	3535	5439.29	<0.001
Model 3: SRL	2564.50**	1365	1.88	0.07	0.065 [0.05, 0.06]	0.85	3026.496	3 vs. 1	2643	3965.71	<0.001

** *p* < 0.001.

**FIGURE 3:**
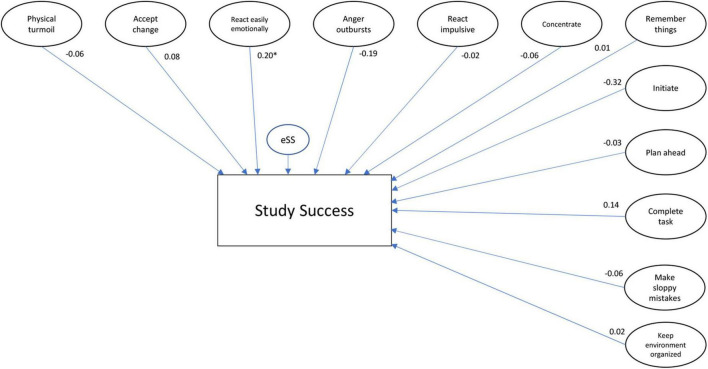
(Self-reported) EF model, including all the latent variables of EF, standardized betas, and *p*-values. *p < 0.05.

**FIGURE 4:**
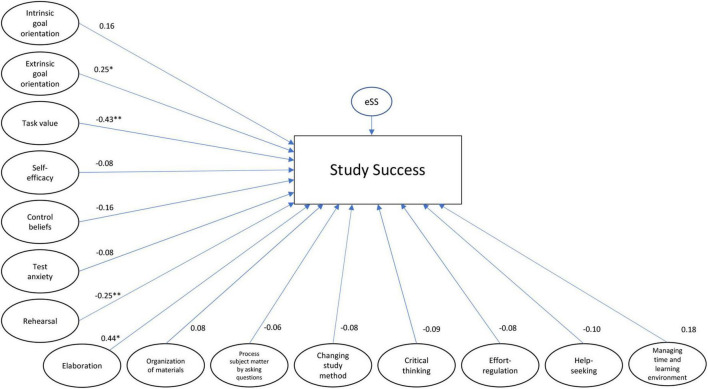
(Self-reported) SRL model, including all the latent variables of SRL, standardized betas, and *p*-values. *p < 0.05; **p < 0.01.

To explore if the combination of EF and SRL explains the variance in the number of credits better than EF and SRL separately, Models 2 and 3 were tested (respectively, Figures 3, 4). The model with the EF variables provided a good fit to the data (χ^2^/df = 1.47; SRMR = 0.06; RMSEA = 0.04; CFI = 0.93), explaining 19.8% of the variance in obtained credits. The model with the SRL variables explained 22.9% of the variance in obtained credits and had a worse fit than the model of the EF, but can be considered a sufficient fit to the data (χ^2^/df = 1.88; SRMR = 0.07; RMSEA = 0.05; CFI = 0.85), again except the CFI threshold. Chi-square difference tests showed that both the EF and SRL models differ significantly from the total model (respectively EF model: χ^2^diff = 5,439.29; df = 3,535; *p* < 0.001 and SRL model: χ^2^diff = 3,965.71; df = 2,643; *p* < 0.001), indicating that the combination of EF and SRL better explains the variance in obtained credits than EF and SRL separately.

In conclusion, the total model explains the most variance (39.8%) in the obtained credits.

### 3.4 Impact of the COVID-19 pandemic on executive functions and self-regulated learning

We used Pearson’s correlation method to calculate the mean score on the question assessing the impact of the COVID-19 pandemic (*M* = 1.74; SD = 0.78) with the index scores of the BRIEF-A (EF) and MSLQ (SRL). The data showed weak, significant correlations between the answer to this question and EF metacognition (*r* = −0.21; *p* < 0.001), SRL motivational strategies (*r* = 0.18; *p* = 0.001), and SRL learning strategies (*r* = 0.15; *p* = 0.007). These results imply that the more the COVID period has led students to have a more pessimistic attitude toward their study process, the more EF metacognition problems were reported, and the fewer SRL strategies were used.

## 4 Discussion

This study sought to investigate the added value of including EFs and SRL in predicting study success after one academic year among higher education students. We explored (1) the relationship between EF and SRL the relationship between EF and study success and SRL and study success, and we hypothesized that (2) the combination of self-reported EF and SRL would explain differences in study success better than separately. First, Regarding the relationships between the constructs our findings show that EF and SRL are correlated. This is consistent with previous studies demonstrating associations between EF and SRL among high school students (Effeney et al., 2013; Rutherford et al., 2018) and university students (Garner, 2009; Follmer and Sperling, 2016). The weak to moderate correlations between EF and SRL makes it clear that these concepts partially overlap but are not the same (Garner, 2009). In addition, EF and SRL both correlate – partially and weakly – with study success, indicating a trend of more EF problems or less SRL comes with fewer credits earned after one school year, in line with studies such as those by Baars et al. (2015) and Ramos-Galarza et al. (2020) for EF, and Li et al. (2018) and Moghadari-Koosha et al. (2020) for SRL.

Second, to better understand the influence of EF and SRL combined on the differences in study success, we compared the imposed models separately and combined. In line with our hypothesis, EF and SRL combined explained the variance in study success after one academic year better than EF and SRL separately. This indicates that a student who performs poorly on EF will likely demonstrate less effective SRL and likely have less study success. Similar results were found by Musso et al. (2019), although they used task-based EF measurements, whereas we used self-reported EF. Even though more research is needed, Musso’s and our findings indicate that combining EF and SRL is vital for the learning processes. A student with more developed EF strategies can reflect on, choose from, or integrate rules where appropriate (Moran and Gardner, 2018, pp. 29–56) and thus be better able to self-regulate their learning and achieve more success.

The current study has some important strengths, such as the empirical confirmation of the need for integration of two theoretical models relevant to education and study success, with the use of proven valid and reliable instruments and the use of SEM to test the models while better accounting for measurement error (Tomarken and Waller, 2005).

On the other hand, this study has a few limitations. First, a non-probabilistic sample was used, namely students of Applied Psychology, which limits the generalization of the results to other groups of students or young adults. Future research could include students from different studies and levels as a more representative sample of young adult learners.

Second, self-reporting measurements were used, which have apparent advantages, such as surveying a large population without much effort and high ecological validity (Barkley and Fischer, 2011). However, a known pitfall with self-reporting is that students may (un)consciously fill out the questionnaires differently than they would show in observed behavior (e.g., McDonald, 2008; Demetriou et al., 2015).

Particularly, self-reports of cognitive abilities are sensitive to response bias and psychological factors, such as depression, anxiety, or chronic pain. For instance, Schwartz et al. (2020) found that self-reporting EF with the BRIEF-A probably measured emotional distress over executive dysfunction. However, they argued that this could be the case in specific samples such as theirs, namely middle-aged veterans who all showed intact EF and experienced heightened psychiatric distress. Abeare et al. (2021) demonstrated inconsistent results to the conclusion of Schwartz et al. (2020), suggesting a more plausible explanation that “non-credible presentation manifests as extreme levels of symptoms on the BRIEF-A-SR- and self-report inventories in general” (Abeare et al., 2021, p. 9). Additionally, a reasonable number of studies have shown that the BRIEF-A can validly measure EF in various target groups such as deaf and hearing students (Hauser et al., 2013), students and procrastination (Rabin et al., 2011), and depression within students (Mohammadnia et al., 2022).

Nevertheless, both Schwartz et al. (2020) and Abeare et al. (2021) suggest that on the validity scale “negativity” a cut-off score of 4 (instead of ≥6) is more representative (i.e., essentially a frequency count of the extreme self-ratings on 10 items of the BRIEF-A). In our study, this would imply that 10 more students should have been disregarded as outliers. However, considering this small number of students, we do not expect different outcomes. To gain insight into the impact of assessment mode on outcomes, research is needed that includes both self-report measures of EF and SRL and objective measures, such as neuropsychological tests for EF or learning analytics for SRL (Yamada et al., 2017). Additionally, measurement instruments that can support screening for non-credible symptom reports can be used (Abeare et al., 2021), such as the MMPI-2 (Schwartz et al., 2020).

Subsequently, a CFI (just) below 0.9 indicates a reasonable but not good fit of the model with the dependent variable. That is, as argued, if the CFI scale is considered as a continuum and not, as is often incorrect, as a dichotomous scale. If our model had had a higher CFI, it would be easier to make statements about the relationship between the variables in the model and the outcome measure. However, this does not alter the fact that the correlations between the various SRL scales and the EF scales with study success have been reliably established. The lower CFI mainly concerns correlations between these (sub)scales, making it more difficult to see what their unique contribution is to study success. Further research will be required to investigate this further.

A final limitation might have been that this study was conducted during the COVID-19 pandemic. Research demonstrates that the lockdowns and other restrictive regulations impacted students’ lives considerably (e.g., Copeland et al., 2021; Ihm et al., 2021), and therefore we investigated the self-reported impact of COVID on how students completed the questionnaires. We found that this period negatively related to how students experience their EF and SRL. This was especially true for students reporting severe EF problems, which implies that the results might be colored because assessments were conducted during the pandemic. Appelhans et al. (2021) found a similar result: young adults with preexisting EF deficits have shown increased unhealthy behavior since COVID-19. We think this period especially challenged students’ EF because of the constant flow of new and complex issues they encountered. Nevertheless, although students’ response patterns might have deviated a bit due to COVID-19, we think that, in light of previous research, patterns would have been the same when assessed in regular times. However, it might be valuable to repeat the study in non-pandemic times.

Future research could further explore how EF and SRL impact study success in theory and practice. One aspect is that a large part of the variance in study success is still unaccounted for, and future research could focus on finding additional answers, for example, in personal and contextual regulatory factors, such as studied by De la Fuente et al. (2022) and Pachón-Basallo et al. (2022).

Another aspect is that EF and SRL have different yet complementary conceptual lenses on how students learn and achieve success [such as Dinsmore et al. (2008) suggest for metacognition and SRL]. Although our study is not about the conceptual lens of EF and SRL, further research into how we can learn from both ways of looking at things to understand student study behavior is desirable since the results confirm that, taken together, they better predict study success even though they do not measure the same thing. The findings of this study can be used to motivate improving learning environments in higher education. Since EF and SRL combined better explain the differences in study success, it makes sense to look at the available EF tools to expand the educational professional’s toolbox beyond the already available SRL tools (e.g., Theobald, 2021). Providing education of EF in addition to SRL probably increases the levels of success in students. Furthermore, metacognitive knowledge about SRL and EF leads to more motivation to use the learned strategies correctly (e.g., Veenman, 2011; Follmer, 2021). Additionally, in the design of (blended) learning environments, educational professionals can build a certain degree of adaptivity when considering different levels of students’ EF, knowing that individual differences are significant. For example, regarding problems with task initiation, one can think of a lesson structure with more intermediate moments during which a student can ask a supervisor for help, more formative tests, or additional (warm-up) assignments in a module that support and encourage the start-up. This way of working is not new and also falls under the intersection of educational science, psychology, and neuroscience, also called neuroeducation (e.g., Jolles and Jolles, 2021; Willingham, 2023).

## 5 Conclusion

In conclusion, this study highlights that while EF and SRL cohere and are related to study success, they do not measure the same. This is also reflected in that each separately explains less variance in study success than taken together. Nonetheless, combined they provide more information about how student achieve study success. Generally, a student reporting EF deficits will likely report less effective SRL and achieve less study success in the long-term. This suggests that attention to EF alongside SRL in education is justified and valuable. Future theoretical research on both the working mechanisms of EF and SRL is needed, as well as the more practical application of the knowledge associated with EF and SRL.

## Data availability statement

The raw data supporting the conclusions of this article will be made available by the authors, without undue reservation.

## Ethics statement

The studies involving humans were approved by Ellis van Dooren-Wissebom, chairperson of the LLM Saxion Ethical Advice Committee (SEAC). The studies were conducted in accordance with the local legislation and institutional requirements. Written informed consent for participation in this study was provided by the participants.

## Author contributions

DM secured funding for the study, conceptualized the study, oversaw, and conducted the data collection, analyzed the data, and led the writing of the manuscript. MS-D managed the data collection in SPSS and contributed to drafting the method and result sections of the manuscript. JD supervised the design, data collection and analyses, and manuscript writing. JF supervised the writing of the manuscript. AJ provided feedback on the latest versions of the article. All authors contributed to the article and approved the submitted version.
