# Supplementary material for: The combined value of executive functions and self-regulated learning to predict differences in study success among higher education students
Source: Front Psychol. 2023 Nov 18;14:1229518. doi: 10.3389/fpsyg.2023.1229518 (PMC10795759; doi:10.3389/fpsyg.2023.1229518)
Supplement: Supplementary file 1 [file Table_1.docx]

Supplementary Table 1. Latent variables of the BRIEF-A and MSLQ, and included items.

| **Latent variables** | **Included items*** | **Cronbach’s α /  Spearman Brown Coefficient**** |  |
| --- | --- | --- | --- |
| **BRIEF-A** |  |  |  |
| Physical turmoil | INH 5, 16 | .74** |  |
| Accept change | SH 8, 44, 61 | .69 |  |
| React easily emotionally | EM 12, 19, 28, 33, 42, 51, 69, 72 | .91 |  |
| Anger outbursts | EM 1, 51 | .64** |  |
| React impulsive | SM 50, 64, 70 | .67 |  |
| Concentrate | WM 4, 35 | .73** |  |
| Remember things | WM 11, 17, 46, 56, 68 | .73 |  |
| Initiate | INI 6, 20, 25, 49, 53 | .75 |  |
| Plan ahead | PL 34, 63 | .79** |  |
| Complete task | TM 52, 75 | .77** |  |
| Make sloppy mistakes | TM 2, 41 | .68** |  |
| Keep environment organized | OM 7, 40, 60, 74 | .83 |  |
| **MSLQ** |  |  |  |
| Intrinsic goal orientation | IGO 1, 2, 3, 4 | .69 |  |
| Extrinsic goal orientation | EGO 1, 2, 3, 4 | .69 |  |
| Task value | TV 1, 2, 3, 4, 5, 6 | .87 |  |
| Self-efficacy | SE 1, 2, 3, 4, 5, 6, 7, 8 | .93 |  |
| Control beliefs | CB 2, 4 | .69** |  |
| Test anxiety | TA 1, 3, 4, 5 | .86 |  |
| Rehearsal | REH 3, 4 | .85** |  |
| Elaboration | ELA 1, 2, 3, 4, 6 | .67 |  |
| Organization of materials | ORG 1, 2, 3 | .70 |  |
| Process subject matter by asking questions | MET 2, 6, 9, 12 | .74 |  |
| Changing study method | MET 4, 7 | .68** |  |
| Critical thinking | CRI 1, 2, 5 | .82 |  |
| Effort regulation | EFF 1, 2, 3, 4 | .72 |  |
| Help-seeking | HS 1, 2, 3, 4 | .73 |  |
| Managing time and learning environment | MTL 1, 4 | .65** |  |

* The numbers correspond with the item numbers in supplement 3 and 4.
