# Supplementary material for: The combined value of executive functions and self-regulated learning to predict differences in study success among higher education students
Source: Front Psychol. 2023 Nov 18;14:1229518. doi: 10.3389/fpsyg.2023.1229518 (PMC10795759; doi:10.3389/fpsyg.2023.1229518)
Supplement: Supplementary file 2 [file Table_2.docx]

Supplementary Table 2. Original scales of the BRIEF-A and MSLQ, and removed items.

| **Original scales** | **Removed items*** |
| --- | --- |
| BRIEF-A |  |
| Inhibition | 29, 36, 55, 58, 73 |
| Shift | 22, 32, 67 |
| Emotional control | - |
| Self-monitor | 13, 23, 37 |
| Working memory | - |
| Initiate | 14, 45, 62 |
| Plan | 9, 15, 21, 39, 47, 66, 71 |
| Task-monitor | 18, 24 |
| Organization of materials | 3, 30, 31, 65 |
| MSLQ |  |
| Intrinsic goal orientation | - |
| Extrinsic goal orientation | - |
| Task value | - |
| Self-efficacy | - |
| Control beliefs | 1, 3 |
| Test anxiety | 2 |
| Rehearsal | 1, 2 |
| Elaboration | 5 |
| Organization | 4 |
| Metacognitive Self-Regulation | 1, 3, 5, 8, 10, 11 |
| Critical thinking | 3, 4 |
| Effort regulation | - |
| Help-seeking | - |
| Managing time and learning environment | 2, 3, 5, 6, 7, 8 |

* The numbers correspond with the item numbers in supplementary Tables 3 and 4.
