# Supplementary material for: The combined value of executive functions and self-regulated learning to predict differences in study success among higher education students
Source: Front Psychol. 2023 Nov 18;14:1229518. doi: 10.3389/fpsyg.2023.1229518 (PMC10795759; doi:10.3389/fpsyg.2023.1229518)
Supplement: Supplementary file 3 [file Table_3.docx]

Supplementary Table 3. Subscales and items BRIEF-A

| **Subscale** | **Items English translation** | **Dutch version** |
| --- | --- | --- |
| Inhibit (INH) | 5. I drum my fingers or wiggle my legs. | Ik trommel met mijn vingers of wiebel met mijn benen. |
|  | 16. I have difficulty staying still. | Ik heb moeite om stil te blijven zitten. |
|  | 29. I find it hard to wait my turn. | Ik vind het moeilijk om op mijn beurt te wachten. |
|  | 36. I make inappropriate sexual comments. | Ik maak ongepaste seksueel getinte opmerkingen. |
|  | 43. I make decisions that get me into trouble (legally, financially, socially). | k neem beslissingen die me in de problemen brengen (wettelijk, financieel, sociaal). |
|  | 55. People say I am easily distracted. | Men zegt dat ik snel afgeleid ben. |
|  | 58. I rattle things off. | Ik raffel dingen af. |
|  | 73. I am impulsive. | Ik ben impulsief. |
| Shift (SH) | 8. I have difficulty making the transition from one activity or task to another. | Ik heb moeite om de overgang van de ene activiteit of taak naar de andere te maken. |
|  | 22. I have difficulty accepting that there are different ways to solve problems with work, with friends or with tasks. | Ik heb moeite te accepteren dat er verschillende manieren zijn om problemen met werk, met vrienden of met taken op te lossen. |
|  | 32. I struggle to think of another solution to a problem when I am stuck. | Ik heb moeite om een andere oplossing voor een probleem te bedenken als ik ben vastgelopen. |
|  | 44. I have a hard time coping with change. | Ik kan slecht tegen veranderingen. |
| Emotional control (EC) | 61. I get upset at unexpected changes in my daily routine. | Ik raak van slag bij onverwachte veranderingen in mijn dagelijkse routine. |
|  | 67. I don't get over problems easily. | Ik zet me niet gemakkelijk over problemen heen. |
|  | 1. I have outbursts of anger. | Ik heb woede-uitbarstingen. |
|  | 12. I overreact emotionally. | Ik reageer overdreven emotioneel. |
|  | 19. I can react very emotionally to small things. | Ik kan om kleine dingen zeer emotioneel reageren. |
|  | 28. I react more emotionally to situations than my friends. | Ik reageer meer emotioneel op situaties dan mijn vrienden. |
|  | 33. I overreact to small problems. | Ik reageer overdreven op kleine problemen. |
|  | 42. I get emotionally upset easily. | Ik raak emotioneel snel overstuur. |
|  | 51. My angry outbursts are intense but quickly over. | Mijn woede-uitbarstingen zijn hevig, maar snel weer voorbij. |
|  | 57. People say I get emotional too quickly. | Men zegt dat ik te snel emotioneel ben. |
|  | 69. My temper turns quickly. | Mijn humeur slaat snel om. |
|  | 72. Little things upset me quickly or easily. | Ik raak door kleine dingen snel of gemakkelijk van streek. |
| Self-monitor (SM) | 13. If I cause others to feel bad or get angry, I don't notice until it's already too late. | Als ik ervoor zorg dat anderen zich slecht voelen of boos worden, merk ik dat pas op als het al te laat is. |
|  | 23. I talk at the wrong times. | Ik praat op verkeerde momenten. |
|  | 37. When people seem upset by me I don't understand why. | Als mensen van streek lijken door mij snap ik niet waarom. |
|  | 50. I say things without thinking | Ik zeg dingen zonder na te denken |
|  | 64. People say I don't think before I do something. | Men zegt dat ik niet nadenk voor ik iets doe. |
|  | 70. I don't think about the consequences before I do something. | Ik denk niet na over de gevolgen voordat ik iets doe. |
| Initiate (INI) | 6. I need to be reminded to start a task, even if I am ready to to perform the task. | Ik moet eraan herinnerd worden om met een taak te beginnen, zelfs als ik bereid ben om die taak uit te voeren. |
|  | 14. I struggle to get ready for the day. | Ik heb moeite om me klaar te maken voor de dag. |
|  | 20. I just hang out at home. | Ik hang thuis maar wat rond. |
|  | 25. I have trouble getting to work independently. | Ik heb moeite om zelfstandig aan de slag te gaan. |
|  | 45. I find it hard to be enthusiastic about things. | Ik vind het moeilijk om enthousiast over dingen te zijn. |
|  | 49. I have difficulty starting tasks. | Ik heb moeite om aan taken te beginnen. |
|  | 53. I only start things (e.g. assignments, chores, tasks) at the last minute. | Ik begin pas op het nippertje aan dingen (bijvoorbeeld opdrachten, karweitjes, taken). |
|  | 62. I have difficulty thinking of things to do in my free time. | Ik heb moeite om dingen te bedenken die ik in mijn vrije tijd kan doen. |
| Working memory (WM) | 4. I have difficulty concentrating on tasks (e.g. chores, reading or work). | Ik heb moeite om me te concentreren op taken (bijvoorbeeld karweitjes, lezen of werk). |
|  | 11. I have difficulty with tasks or tasks that require more than one step. | Ik heb moeite met opdrachten of taken die meer dan één stap vereisen. |
|  | 17. Halfway through an activity, I forget what I was doing. | Halverwege een activiteit vergeet ik wat ik aan het doen was. |
|  | 26. I have difficulty staying on the same topic while talking. | Ik heb moeite om tijdens het praten bij hetzelfde onderwerp te blijven. |
|  | 35. I can only concentrate for a short time. | Ik kan me maar kort concentreren. |
|  | 46. I forget directions quickly. | Ik vergeet aanwijzingen snel. |
|  | 56. I have trouble remembering things even for a few minutes (such as directions, phone numbers). | Ik heb moeite om dingen te onthouden, zelfs voor een paar minuten (zoals aanwijzingen, telefoonnummers). |
|  | 68. I have difficulty doing more than one thing at a time. | Ik heb moeite om meer dan één ding tegelijk te doen. |
| Plan (PL) | 9. I get overwhelmed by big tasks. | Ik word overweldigd door grote taken. |
|  | 15. I find it difficult to prioritise activities. | Ik vind het lastig om prioriteiten te stellen bij activiteiten. |
|  | 21. I start tasks (e.g. cooking, projects) without having the right materials available have them. | Ik begin aan taken (bijvoorbeeld koken, projecten) zonder de juiste materialen ter beschikking te hebben. |
|  | 34. I don't plan future activities in advance. | Ik plan toekomstige activiteiten niet van tevoren. |
|  | 39. I set unrealistic goals. | Ik stel onrealistische doelen. |
|  | 47. I have good ideas but can't get them down on paper. | Ik heb goede ideeën, maar krijg ze niet op papier. |
|  | 54. I struggle to complete tasks independently. | Ik heb moeite om taken zelfstandig af te maken. |
|  | 63. I don't plan tasks ahead. | Ik plan taken niet vooruit. |
|  | 66. I have difficulty organising activities. | Ik heb moeite bij het organiseren van activiteiten. |
|  | 71. I have difficulty organising my work. | Ik heb moeite om mijn werk te organiseren. |
| Task-monitor (TM) | 2. I make carelessness mistakes when making tasks. | Ik maak slordigheidsfouten bij het maken van taken. |
|  | 18. I don't check my work for errors. | Ik controleer mijn werk niet op fouten. |
|  | 24. I misjudge how difficult or easy tasks will be. | Ik schat verkeerd in hoe moeilijk of gemakkelijk taken zullen zijn. |
|  | 41. I make sloppy mistakes. | Ik maak slordigheidsfoutjes. |
|  | 52. I have difficulty completing tasks (e.g. chores, work). | Ik heb moeite om taken (bijvoorbeeld karweitjes, werk) af te maken. |
|  | 75. I have problems finishing my work. | Ik heb problemen om mijn werk af te maken. |
| Organization of materials (OM) | 3. I can't organise well. | Ik kan niet goed organiseren. |
|  | 7. I have a messy closet. | Ik heb een rommelige kast. |
|  | 30. People say I can't organise well. | Men zegt dat ik niet goed kan organiseren. |
|  | 31. I lose things (e.g. keys, money, wallet, homework, etc.). | Ik raak dingen kwijt (bijvoorbeeld sleutels, geld, portemonnee, huiswerk, etc.). |
|  | 40. I leave the bathroom messy. | Ik laat de badkamer rommelig achter. |
|  | 60. I leave my room or house messy. | Ik laat mijn kamer of huis rommelig achter. |
|  | 65. I have trouble finding things in my room, closet or desk. | Ik heb moeite om dingen te vinden in mijn kamer, kast of bureau. |
|  | 74. I don't tidy up my things. | Ik ruim mijn spullen niet op. |
