# Supplementary material for: The combined value of executive functions and self-regulated learning to predict differences in study success among higher education students
Source: Front Psychol. 2023 Nov 18;14:1229518. doi: 10.3389/fpsyg.2023.1229518 (PMC10795759; doi:10.3389/fpsyg.2023.1229518)
Supplement: Supplementary file 4 [file Table_4.docx]

Supplementary Table 4. Subscales and items MSLQ

| **Subscale** | **Items original version** | **Dutch translation** |
| --- | --- | --- |
| Intrinsic goal orientation (IGO) | 1. With this module, I would like challenging study material so that I can learn new things. | Bij deze module wil ik graag uitdagend studiemateriaal zodat ik nieuwe dingen kan leren. |
|  | 2. For a module like this, I prefer study material that stimulates my curiosity, even if the material is difficult. | Bij een module als deze heb ik het liefst studiemateriaal dat mijn nieuwsgierigheid prikkelt, ook al is de stof moeilijk. |
|  | 3. It gives me great satisfaction when I try to understand the material of this module as well as I can. | Het geeft me veel voldoening als ik de leerstof van deze module zo goed mogelijk probeer te begrijpen. |
|  | 4. If I get the chance in this module, I choose the assignments that I learn something from, even if they do not guarantee a good grade. | Als ik de kans krijg in deze module, kies ik de opdrachten waar ik iets van leer, zelfs als die geen garantie geven op een goed cijfer. |
| Extrinsic goal orientation (EGO) | 1. Getting a good grade in this module gives me the most satisfaction now. | Een goed cijfer halen voor deze module geeft mij nu de meeste voldoening. |
|  | 2. I do aim to get as high a grade as possible on average and therefore, in this module I would like to get a good grade. | Ik ben er wel op uit gemiddeld een zo hoog mogelijk cijfer te halen en daarom wil ik bij deze module graag een goed cijfer halen. |
|  | 3. I would like to get higher marks than most of my fellow students. | Ik wil graag hogere cijfers halen dan de meesten van mijn medestudenten. |
|  | 4. I would like to be good at this module to show others like my family, friends, or employer that I am successful. | Ik wil graag goed zijn in deze module om anderen zoals mijn familie, vrienden, of werkgever te laten zien dat ik succesvol ben. |
| Task value (TV) | 1. I think what I learn in this module, I can also use in other modules. | Ik denk dat wat ik leer tijdens deze module, ook kan gebruiken bij andere modules. |
|  | 2. I think it is important for me to learn the material in these modules. | Ik vind het belangrijk dat ik de lesstof van deze modules leer. |
|  | 3. I am very interested in the specific content of these modules. | Ik ben erg geïnteresseerd in de specifieke inhoud van deze modules. |
|  | 4. The teaching material of this module is useful for me to learn. | Het lesmateriaal van deze module is nuttig voor mij om te leren. |
|  | 5. The topics of this module appeal to me. | De onderwerpen van deze module spreken mij aan. |
|  | 6. It is important for me to understand the topics of this module. | Ik vind het belangrijk dat ik de onderwerpen van deze module begrijp. |
| Self-efficacy (SE) | 1. I think I will get very good marks for this module. | Ik denk dat ik zeer goede cijfers ga halen voor deze module. |
|  | 2. I am sure I can understand the most difficult parts I have to study in this module. understand. | Ik weet zeker dat ik de moeilijkste onderdelen die ik moet bestuderen bij deze module, kan begrijpen. |
|  | 3. I am confident that I can learn the basic concepts of this module. | Ik heb er vertrouwen in dat ik de basisconcepten van deze module kan leren. |
|  | 4. I am confident that I can understand. | Ik ben er zeker van dat ik de meest moeilijke onderwerpen die de docent voorlegt wel kan begrijpen. |
|  | 5. I am confident that I can produce good work for the assignments and test associated with the module. | Ik heb er vertrouwen in dat ik goed werk kan leveren voor de opdrachten en de toets die horen bij de module. |
|  | 6. I expect to pass this module well. | Ik verwacht deze module goed te doorlopen. |
|  | 7. I am confident that I will master the skills to be learned within this module. | Ik weet zeker dat ik de te leren vaardigheden binnen deze module onder de knie ga krijgen. |
|  | 8. Looking at the difficulty of the material taught, the teachers and my own ability, I expect to achieve good results for this module. | Als ik kijk naar de moeilijkheidsgraad van de lesstof, de docenten en naar mijn eigen kunnen, dan verwacht ik goede resultaten te behalen voor deze module. |
| Control beliefs (CB) | 1. If I study in the right way, I will master the material in this module. | Als ik op de goede manier studeer, krijg ik de stof van deze module wel onder de knie. |
|  | 2. If I fail this module, it is primarily down to myself . | Als ik deze module niet haal, ligt dat in de eerste plaats aan mezelf . |
|  | 3. If I make enough effort, I will manage to understand the material. | Als ik mij genoeg inspan, lukt het mij wel de leerstof te begrijpen. |
|  | 4. If I don't understand the material, it's because I haven't made enough effort. | Als ik de stof niet begrijp komt dat omdat ik te weinig moeite heb gedaan. |
| Test anxiety (TA) | 1. When I take a test, I think about how badly I am doing compared to other students. | Wanneer ik een toets maak, dan denk ik eraan hoe slecht ik het doe in vergelijking met andere studenten. |
|  | 2. When I'm working on a test question, I think about the other questions to which I don't know. | Wanneer ik met een toetsvraag bezig ben, denk ik aan de andere vragen waarop ik het antwoord niet weet. |
|  | 3. When I take a test, I think about what happens if I fail it. | Als ik een toets maak, denk ik aan wat er gebeurt als ik hem niet haal. |
|  | 4. While taking a test, I feel uncomfortable and stressed. | Tijdens het maken van een toets heb ik een ongemakkelijk en gestrest gevoel. |
|  | 5. I suffer from an increased heart rate when taking a test. | Ik heb last van een verhoogde hartslag wanneer ik een toets maak. |
| Rehearsal (REH) | 1. I repeat the most important parts of the study material out loud. | Ik herhaal de belangrijkste onderdelen van de studiestof hardop. |
|  | 2. When I study for this module, I review my notes and the study material repeatedly. | Als ik voor deze module leer, kijk ik mijn aantekeningen en de leerstof herhaaldelijk door. |
|  | 3. To master the key concepts of the module, I learn definitions from my by heart. | Om de belangrijkste begrippen van de module onder de knie te krijgen leer ik definities uit mijn hoofd. |
|  | 4. I make lists of the important definitions in this module and learn them by heart. | Ik maak lijsten van de belangrijke definities in deze module en leer die uit mijn hoofd. |
| Elaboration (ELA) | 1. As I study for this module, I collect and integrate information from different sources, | Als ik studeer voor deze module verzamel en integreer ik informatie uit verschillende bronnen, |
|  | such as lecture notes, study materials and discussions. | zoals lesaantekeningen, studiematerialen en discussies. |
|  | 2. I try to connect the theory in this module with the theory from other modules. | Ik probeer de theorie in deze module te verbinden met de theorie uit andere modules. |
|  | 3. When I read something for this module, I try to connect what I read with what I already know. | Wanneer ik iets lees voor deze module, probeer ik wat ik lees te verbinden met wat ik al weet. |
|  | 4. When I study for this module, I make summaries of the central points from the | Als ik leer voor deze module maak ik samenvattingen van de centrale punten uit het studiemateriaal en mijn aantekeningen. |
|  | study material and my notes. |  |
|  | 5. I try to understand the subject matter of this module by making connections between what we have to read and the concepts explained during the lecturer's presentations. | Ik probeer de leerstof van deze module te begrijpen door verbanden te leggen tussen wat we moeten lezen en de begrippen die zijn uitgelegd tijdens de presentaties van de docent. |
|  | 6. I try to apply the concepts from the study materials to be studied in other teaching activities such as a presentation or discussion. | Ik probeer de begrippen uit de te bestuderen studiematerialen toe te passen in andere lesactiviteiten zoals een presentatie of een discussie. |
| Organization (ORG) | 1. When reading the module, I make summaries of the main points to organise my thoughts organise my thoughts. | Bij het lezen van de module maak ik overzichten van de belangrijkste punten om mijn gedachten te ordenen. |
|  | 2. When I am learning for this module, I go through what I have read and written down and try to pick out the most important things from it. | Wanneer ik leer voor deze module neem ik door wat ik gelezen en opgeschreven heb en probeer ik het belangrijkste eruit te halen. |
|  | 3. I make diagrams, summaries and tables to organise the material. | Ik maak schema’s, overzichten en tabellen om de stof te ordenen. |
|  | 4. When I study for this module, I go through my notes and make a summary of the key concepts. | Als ik leer voor deze module, loop ik mijn aantekeningen na en maak ik een overzicht van de belangrijkste begrippen. |
| Metacognition (MET) | 1. I often miss important points during a lecture because my mind is elsewhere (R). | Tijdens een college mis ik vaak belangrijke punten omdat ik met mijn gedachten ergens anders ben. (R) |
|  | 2. When I read for this module, I think of questions to keep my attention. | Wanneer ik lees voor deze module, bedenk ik vragen om mijn aandacht erbij te houden. |
|  | 3. If I get confused by something I read, I re-read it and try to get out of it. | Als ik in de war raak van iets dat ik lees, dan lees ik het opnieuw en probeer ik eruit te komen. |
|  | 4. If the study material is difficult to understand, I apply a different strategy of study. | Als het studiemateriaal moeilijk te begrijpen is, pas ik een andere strategie van bestuderen toe. |
|  | 5. Before reading a new text, I take a cursory look at how the text is structured. | Voordat ik een nieuwe tekst ga lezen, bekijk ik eerst vluchtig hoe de tekst is opgebouwd. |
|  | 6. I ask myself questions to make sure I understand the study material. | Ik stel mezelf vragen om er zeker van te zijn dat ik de leerstof begrijp. |
|  | 7. I adapt my study methods to the requirements for the module. | Ik pas mijn studiemethoden aan aan de eisen voor de module. |
| Critical thinking (CRI)  Effort regulation (EFF)  Peerlearning  Help-seeking (HS)  Managing time and learning environment (MTL) | 8. It regularly happens to me that I have read something for this module and I do not understand what it is about (R)  9. Instead of starting reading right away, I think about a topic first and see what I should learn from it.  10. When I'm studying for this module, I find out which concepts I don't understand yet.  11. When I am studying for this module, I set goals for myself to guide my study activities during the quartile.  12. If I find my notes unclear during class, I sort it out immediately after class.  1. I find that with this module I often wonder if I find the things I read or hear convincing.  2. I see this module as a starting point to develop my own vision.  3. About what I am learning, I also try to formulate my own views.  4. As soon as a theory, interpretation or conclusion is presented at this module, I question whether it is well-founded.  5. Every time I read or hear a proposition or conclusion presented at this module, I think about whether you can also claim or conclude something else.  1. I am often too lazy or bored when I study, so I stop before I even finish what I had planned for this module (R).  2. I work hard to perform well even though I don't like what we have to do for this module.  3. If the study material is difficult I give up or I study only the easy parts (R).  4. Even if the study material is boring and uninteresting, I manage to work through until I finish.  5. If I feel too tense or stressed, I consciously relax so that I can learn with good concentrated learning.  1. I sometimes try to explain the subject matter to a friend or classmate, because I then understand it better myself.  2. If I can, I cooperate with other students in making and completing assignments.  3. I regularly make time to discuss the subject matter with a group of fellow students.  1. Even when I struggle to learn, I don't ask others for help (R).  2. If I don't understand definitions, I ask the teacher to clarify them.  3. If I don't understand the material, I ask a fellow student for help.  4. I think of the best fellow student to ask when I need help.  1. I usually sit to learn in a place where I can concentrate.  2. I use my time efficiently in this module.  3. I find it difficult to stick to a schedule (R).  4. I often study in the same place.  5. I make sure I have read everything and done the assignments every week.  6. I attend class regularly.  7. I regularly find that I do not spend enough time on this module because I am busy with other things.  8. I almost never manage to make time to go through my notes and reading materials before a test (R). | Het overkomt me regelmatig dat ik iets heb gelezen voor deze module en dat ik niet begrijp waarover het gaat (R).  In plaats van gelijk te beginnen met lezen, denk ik eerst over een onderwerp na en bekijk wat ik ervan zou moeten leren.  Als ik zit te leren voor deze module, zoek ik uit welke begrippen ik nog niet goed begrijp.  Als ik studeer voor deze module stel ik doelen voor mezelf om richting te geven aan mijn studieactiviteiten tijdens het kwartiel.  Als ik tijdens de les mijn aantekeningen onduidelijk vind, zoek ik het na de les meteen uit.  Ik merk dat ik me bij deze module vaak afvraag of ik de dingen die ik lees of hoor wel overtuigend vind.  Ik zie deze module als een vertrekpunt om mijn eigen visie te ontwikkelen.  Over wat ik aan het leren ben, probeer ik ook mijn eigen denkbeelden te formuleren.  Zodra er een theorie, interpretatie of conclusie wordt gepresenteerd bij deze module, vraag ik me af of die wel goed onderbouwd is.  Steeds als ik bij deze module een stelling of conclusie lees of hoor, bedenk ik of je ook iets anders kunt beweren of concluderen.  Ik ben vaak te lui of te verveeld als ik studeer, zodat ik al stop voordat ik afrond wat ik gepland had voor deze module (R).  Ik werk hard om goed te presteren, ook al vind ik wat we moeten doen voor deze module niet leuk.  Als het studiemateriaal moeilijk is geef ik op of ik bestudeer alleen de makkelijke onderdelen (R).  Zelfs als het studiemateriaal saai en oninteressant is, krijg ik het voor elkaar om door te werken totdat ik klaar ben.  Als ik me te gespannen of gestrest voel, ga ik mij bewust ontspannen zodat ik goed geconcentreerd kan leren.  Ik probeer de leerstof wel eens uit te leggen aan een vriend(in) of klasgenoot, omdat ik het dan zelf beter ga begrijpen.  Als het kan, werk ik met andere studenten samen bij het maken en afronden van de opdrachten.  Ik maak regelmatig tijd vrij om te discussiëren over de leerstof met een groepje medestudenten.  Zelfs als ik moeite heb om te leren, vraag ik geen hulp aan anderen (R).  Als ik definities niet begrijp, vraag ik aan de docent die te verduidelijken.  Als ik de stof niet begrijp vraag ik een medestudent om hulp.  Ik bedenk aan welke medestudent ik het beste iets kan vragen als ik hulp nodig heb.  Meestal zit ik te leren op een plek waar ik me kan concentreren.  Bij deze module ga ik efficiënt met mijn tijd om.  Ik vind het moeilijk me aan een planning te houden (R).  Ik studeer vaak op dezelfde plek.  Ik zorg ervoor dat ik wekelijks alles gelezen heb en de opdrachten heb gemaakt.  Ik ben regelmatig aanwezig bij de les.  Ik merk dat ik regelmatig te weinig tijd besteed aan deze module, omdat ik met andere dingen bezig ben (R).  Het lukt me bijna nooit om tijd vrij te maken om mijn aantekeningen en leesmaterialen door te nemen voor een toets (R). |

Note. A (R) means the item needs to be recoded
